# Recent Advances in Multi- and Hyperspectral Image Analysis

**DOI:** 10.3390/s21186002

**Published:** 2021-09-08

**Authors:** Jakub Nalepa

**Affiliations:** Department of Algorithmics and Software, Faculty of Automatic Control, Electronics and Computer Science, Silesian University of Technology, Akademicka 16, 44-100 Gliwice, Poland; jnalepa@ieee.org

**Keywords:** hyperspectral image analysis, multispectral image analysis, image acquisition, feature extraction, dimensionality reduction, classification, segmentation, unmixing

## Abstract

Current advancements in sensor technology bring new possibilities in multi- and hyperspectral imaging. Real-life use cases which can benefit from such imagery span across various domains, including precision agriculture, chemistry, biology, medicine, land cover applications, management of natural resources, detecting natural disasters, and more. To extract value from such highly dimensional data capturing up to hundreds of spectral bands in the electromagnetic spectrum, researchers have been developing a range of image processing and machine learning analysis pipelines to process these kind of data as efficiently as possible. To this end, multi- or hyperspectral analysis has bloomed and has become an exciting research area which can enable the faster adoption of this technology in practice, also when such algorithms are deployed in hardware-constrained and extreme execution environments; e.g., on-board imaging satellites.

## 1. Introduction

Hyperspectral imaging can capture hundreds of images acquired for narrow and continuous spectral bands across the electromagnetic spectrum [1,2]. As an illustrative example, for the 400–1000 nm spectral range, we can collect approximately 180 single-channel images (one for each band) using a hyperspectral camera with a 3.26 nm spectral resolution, whereas for a sensor operating in the 930–2500 range (with a 5.45 nm spectral resolution), we can capture up to 290 bands. Since the spectral profiles are specific for different materials, exploiting such high-dimensional data can help to determine the characteristics of the objects of interest. Therefore, the analysis of hyperspectral images (HSIs) has attracted research interest in various fields of science and industry, including mineralogy [3], precision agriculture [4,5], medicine [6], chemistry [7], forensics [8], and remote sensing [1,9,10]. On the other hand, multispectral images (MSIs) contain much fewer bands with larger bandwidths [11].

HSIs can be interpreted as data cubes that couple the spatial and spectral information captured for every pixel in the scene. The high dimensionality and volume of hyperspectral data significantly impact the cost and time of transferring such images and make them challenging to analyze and interpret manually [12,13]. There are a plethora of state-of-the-art approaches to automating the hyperspectral data analysis process, and they benefit from a wide spectrum of machine learning, computer vision, and advanced data processing techniques [1]. However, the availability of manually annotated hyperspectral datasets is still limited, and they are often small, not very representative, extremely imbalanced, and noisy; e.g., due to the noise that is intrinsic to the data acquisition itself, especially in the context of satellite imaging. These issues make the supervised machine learning algorithms difficult to apply in emerging hyperspectral image analysis scenarios [14]. Importantly, generating ground-truth image data containing manually-delineated objects of interest is not only user-dependent and error-prone, but also cumbersome and costly, as it requires transferring raw image data for further analysis; e.g., from an imaging satellite or other remote imagers [15]. This issue negatively affects our ability to train well-performing supervised learners for HSI analysis that could benefit from large training samples [16,17]. Additionally, the thorough and fair validation of developed approaches is challenging, as their generalization abilities must be investigated with care in order not to infer over-optimistic (or over-pessimistic) conclusions about their performance [18].

To build end-to-end processing pipelines for MSI/HSI analysis, we need to tackle the aforementioned challenges and issues that are inherent to the characteristics of such data. On the one hand, we suffer from limited ground-truth training samples—to deal with this, we can exploit various data augmentation techniques to increase the size of training sets (if they exist) [19]. On the other hand, the high dimensionality of such imagery, especially HSI, may easily lead to the curse of dimensionality and high levels of data redundancy [20]. To this end, the research community has been developing both band selection and feature extraction algorithms that are designed to effectively deal with HSIs [21]. The former algorithms can help us to maintain the interpretability of the reduced image data, as only the most informative bands survive the band selection process. Extracting new features through feature extraction (also referred to as feature learning) enables us to elaborate low-dimensional embedding spaces, in which the physical meaning of specific bands is lost, and such features may be difficult to interpret. There are, however, well-established approaches for this task that have been shown to be very effective and include both hand-crafted [22] and deep learning [23,24] feature extractors.

Once the original MSI/HSI is appropriately pre-processed, it commonly undergoes segmentation, which is the process of finding coherent regions of similar characteristics. Here, segmentation involves classification, as we often assign class labels to specific pixels of the input image. The approaches for these tasks are split into classical machine learning and deep learning techniques. The former algorithms utilize hand-crafted feature extractors, whereas deep learning algorithms benefit from automated representation learning and therefore do not require feature engineering. The results of MSI/HSI segmentation—which is virtually always an intermediate processing step in the analysis chain—directly influence the next steps that are aimed at extracting specific value from the input data. Here, we may be interested in various use cases that encompass, among others, object tracking in MSI/HSI [25], scene understanding [26], or change detection, which additionally incorporates the temporal aspect of multiple images acquired for the very same scene in different time points [27]. Thus, building high-quality classification and segmentation algorithms is of paramount importance in the field, and a multitude of techniques are continuously emerging.

Finally, the hyperspectral pixel size, together with the spatial resolution of an HSI, can substantially vary across applications and specific imagers. In remote sensing, where the image data are captured on-board a satellite, the corresponding ground sampling distance, which is the distance between the centers of two neighboring pixels measured on the ground, can easily reach tens of meters. Hence, several materials may be present within each pixel, and quantifying the fractional abundance of a given material in an HSI pixel helps us to understand their actual mixture better. Given high intra-class variability and inter-class similarities [28], developing effective algorithms for this task, referred to as hyperspectral unmixing [29], is another important area in the field. Note that gathering ground-truth data for automated hyperspectral unmixing is affected by the very same challenges as in the case of segmentation tasks (unmixing is commonly considered to be a more challenging problem).

Effective analysis of MSI/HSI brings exciting opportunities in various real-life applications in a number of fields. Therefore, we are witnessing an explosion of works that develop and apply machine learning to MSI/HSI analysis. The aim of this Special Issue is to gather and present recent advances in multi- and hyperspectral image analysis. The core themes of this topic cover all steps of the data processing pipeline, from its acquisition to final analysis and understanding. The Special Issue contains eight papers that present the development and verification of exciting technological advances; we briefly discuss these works in Section 2, whereas Section 3 provides the conclusions.

## 2. A Brief Review of the Articles in the Special Issue

Image acquisition, which is a process of capturing an image (or a sequence of images) for a scene of interest, is an initial and crucial step that allows us to gather data for further analysis. The recent technological advances enable us to acquire spectral and polarization image data jointly, and understanding the characteristics of this imagery is pivotal for designing effective image processing routines, which could significantly benefit from these developments. In [30], Courtier et al. investigated different correlations between spectropolarimetric channels. Their experimental study, performed over several specific clusters of materials and reflection types, showed that the inter-channel polarization information appears to be more correlated than for the spectral channels for dielectric materials. The authors, however, highlighted that further investigations are required for metallic objects. This indeed helped to formulate a set of recommendations that could ultimately improve the design process of the algorithms operating with such data, help to design adequate imaging pipelines, or even help to design sensors with better performance.

Fundamentally, different image modalities allow practitioners to perform various image analysis tasks and are commonly characterized by different information entropy. In the work reported by Liu et al. [31], the authors tackled an interesting problem of reconstructing MSI from RGB images, and they utilized cycle generative adversarial networks (CycleGANs) to convert the problem of comparing images from two different domains into a comparison of images from the same domain, presenting a solid theoretical foundation to solve the two distinct domain image translation problems (the authors targeted the problem of the bidirectional translation between RGB images and MSIs). Liu and colleagues pointed out two important challenges that are inherently related to this task and were addressed in their paper. Firstly, RGB images and MSIs belong to two domains with different definitions and synthesis rules, without a direct mapping relation between them. Secondly, it is clear that MSIs have more abundant information than RGB images. Additionally, the authors exploited multilayer perceptrons to substitute convolutional neural nets when implementing the generators to make them simpler and more efficient, cut off two traditional identity losses utilized in CycleGANs to fit the spectral image translation, and introduced two consistency losses to enhance the model training. The authors investigated the performance of the method in their experimental validation over two benchmarks and showed that it is competitive with the state of the art, especially in the context of working with limited training samples. However, it would be interesting to further investigate the abilities of the proposed technique for processing the image data captured above 700 nm and below 400 nm. Finally, benefiting from the recent hardware advances, e.g., GPU or FPGA accelerators, would greatly speed up the algorithm.

As mentioned in the introductory section, the accurate classification and segmentation of HSIs are pivotal tasks; hence, developing better and more robust—e.g., against various kinds of noise—algorithms is important from the practical perspective. In their paper [32], Qu and colleagues proposed the integration of the joint feature extraction framework of spatial and transform domain filtering into their processing chain. Here, the sparse feature and low-rank structure of HSI are utilized to perform transform domain filtering and thus to separate the mixed noise in the image and improve its peak signal-to-noise ratio. Afterwards, the separated feature images are transformed and filtered recursively in a multi-level domain based on the spatial domain. To enhance the spatial–spectral features of the feature image, the strong edges of different objects in the image are preserved and the fine texture structures are removed. Afterwards, the multi-scale entropy ratio-based superpixel segmentation algorithm is utilized to segment the resulting HSI—the image is automatically split into regions with similar characteristics and, subsequently, the dimensionality reduction helps us find the low-dimensional features of the HSI. Then, principal component analysis is performed for each HSI with superpixels in multi-scale, and the spatial information of each HSI is utilized during the segmentation process. Qu et al. executed the experiments over three classical HSI benchmarks, and the results indicate that the suggested analysis pipeline allows high-quality segmentation to be obtained. The authors, however, noted that the algorithm is parameterized; thus, automating the procedure of determining appropriate hyper-parameter values would greatly improve its applicability in real-life settings.

Real-life applications of hyperspectral imaging span across various domains, which is also reflected in our Special Issue. Książek and colleagues focused on the problem of non-invasive substance classification [33], which can play a pivotal role in forensic science, as detecting and classifying various samples in a scene of interest can help improve the investigation; e.g., in the context of blood stain analysis. The authors thoroughly investigated a range of classical machine learning and deep learning algorithms for this task—their experiments included scenarios in which test samples come from the very same HSI and from a different image than the training samples (with the latter experiment being much more challenging and corresponding to real-life settings). Interestingly, Książek et al. presented a model evaluation method based on t-distributed stochastic neighbor embedding and confusion matrix analysis, which allowed for the detection and elimination of some cases of model undertraining. The experiments showed that deep learning can indeed outperform classical machine learning in blood stain classification, especially in the more challenging multiple-image scenario. Blood identification was also tackled by Zulfiqar et al., and the authors suggested a new method based on the visualization of heme-components bands in the 500–700 nm spectral range [34]. In their algorithm, Savitzky Golay filtering is used to highlight the subtle bands of all samples. Then, a variety of classical classifiers, including support vector machines, *k*-nearest neighbors, decision trees, random forests, and artificial neural networks, were trained over the derivative spectrum. The comparative analysis revealed that the proposed technique outperformed several state-of-the-art algorithms. Additionally, the authors highlight important challenges that could be approached in the future work and relate to, among others, limited ground-truth samples, reconstructing old crime scenes (where the aging criteria could also be analyzed in more detail), and extending the dataset with animal blood samples.

There are a number of HSI applications in agriculture. One of these use cases was approached in the work reported by Weksler and colleagues, in which the authors were interested in detecting potassium deficiency and estimating the momentary transpiration rate at early growth stages through the analysis of proximal hyperspectral imaging [35]. It is worth mentioning that potassium is typically supplied to crops in excess throughout the season in order to avoid a deficit that ultimately leads to a reduced crop yield. Additionally, the transpiration rate is a momentary physiological attribute that is indicative of soil water content, a plant’s water requirements, and abiotic stress factors. The authors created a hyperspectral–physiological plant database for the classification of potassium treatments (low, medium, and high), and for the estimation of momentary transpiration rates from hyperspectral images. Additionally, a semi-automated platform equipped with a hyperspectral imager was triggered every hour to capture images of a large array of pepper plants. Afterwards, extreme gradient boosting was utilized to classify plants into the potassium treatments and to estimate the transpiration rates based on the acquired data. The experiments indicated that it is possible to label plants according to potassium treatments based on a remotely measured hyperspectral signal. Additionally, the authors pointed out that the presented ability of estimating transpiration rates for different potassium applications using spectral information can aid in irrigation management and crop yield optimization. Therefore, the proposed system may be helpful in improving the decision-making process during the growing season, particularly at the early stages, when potassium levels can still be corrected to prevent yield loss. An interesting future research pathway that originates from this work includes targeting other field scenarios to further support practical agronomic decision making.

In the paper presented by Sun et al. [36], we can find an example of exploiting the remotely sensed images (panchromatic and MSI) in urban applications. Here, the imagery captured on-board the Gaofen-2 mission was used to analyze the quantity and spatial distribution of blue steel roofs in the Nanhai district, Foshan (including the towns of Shishan, Guicheng, Dali, and Lishui), which is an important manufacturing industry base of China. As indicated by the authors, blue steel panels have been widely used in roof construction in many inefficient industrial areas (factories and warehouses); hence, the distribution of blue steel panels can reflect the industrial structure and economic development of a specific area. Because of that, detecting such objects may become important for the preliminary assessment of inefficient industrial areas and is one of the key elements for quantifying environmental issues, such as urban heat islands. The authors exploited a semantic deep learning segmentation architecture for extracting the blue steel roof information from the scene of interest and highlighted its high accuracy. Interestingly, Sun and colleagues found out that the blue steel roof areas were positively correlated with some economic factors. Therefore, these roofs might serve as an indicator for inefficient industrial areas in regional planning and show their environmental and socio-economic significance.

Finally, in the work reported by Dolet et al. [37], multispectral photoacoustic images were utilized to estimate the concentration of chromophores. In this study, the unmixing techniques were built around two important steps, which were the automatic extraction of the reference spectrum of each pure chromophore (this step was accomplished by various approaches) and the abundance calculation of each pure chromophore from the estimated reference spectra. The authors demonstrated that—for non-biological tissues and using the Vevo LAZR acquisition system—the suggested procedure provides accurate estimates for dilutions and mixtures. The sum-to-one constraint imposed by the fully constrained least-square algorithm embedded in the processing pipeline is, however, a fairly strong assumption, particularly when dilutions are imaged. To relax this constraint, a shadow endmember was introduced by Dolet et al. The approaches were tested on a mouse tumor, but the ground truth of the oxygenation rate in the imaged tumor was not known. This posed an interesting challenge of confronting the investigated algorithms. The authors pointed out several exciting research pathways that could indeed increase the robustness of the results reported in the paper even further.

## 3. Conclusions

Multi- and hyperspectral image processing using machine learning and advanced data analysis has become an important research area due to the numerous challenges that need to be effectively faced before such imaging techniques can be robustly employed in emerging real-life use cases that span across various fields of engineering and industry. In this Special Issue, we presented eight papers that summarize a variety of recent technological advances and present different perspectives of researchers with various scientific background sand experience, specializing in artificial intelligence, remote sensing, environmental and Earth sciences, software engineering, or mathematics.

We believe that the presented papers will inspire other researchers to conduct further work and therefore will serve as a solid foundation for broader research and development in the exciting area of multi- and hyperspectral image processing.

## Data Availability

For the data availability statements, please refer to specific articles in this Special Issue.

## Abbreviations

The following abbreviations are used in this manuscript:

FPGA	Field programmable gate array
GAN	Generative adversarial network
GPU	Graphics processing unit
HSI	Hyperspectral image
MSI	Multispectral image
RGB	Red Green Blue [image]
